# Retroperitoneal fibrosis and bilateral hydronephrosis secondary to gastric cancer: A case report

**DOI:** 10.1097/MD.0000000000043826

**Published:** 2025-08-22

**Authors:** Jiwon Kim, Moon Hyung Lee, Su Bee Park

**Affiliations:** a Department of Medicine, Graduate School, Kyung Hee University, Seoul, Republic of Korea; b Department of Internal Medicine, Kyung Hee University Hospital at Gang Dong, Kyung Hee University College of Medicine, Seoul, Republic of Korea.

**Keywords:** bilateral hydronephrosis, case report, gastric cancer, malignancy-associated fibrosis, retroperitoneal fibrosis, ureteral obstruction

## Abstract

**Rationale:**

Retroperitoneal fibrosis (RPF) is a rare fibroinflammatory condition that can lead to ureteral obstruction and renal dysfunction. While most cases are idiopathic, secondary causes including malignancies should be considered. RPF secondary to gastric cancer is extremely rare, with only a few cases reported.

**Patient concerns:**

A 56-year-old woman presented with generalized edema, decreased urine output, and nausea that had begun 7 days prior. Laboratory tests revealed acute kidney injury, and imaging showed bilateral hydronephrosis.

**Diagnoses:**

Further evaluation through abdominal-pelvic computed tomography and esophagogastroduodenoscopy revealed a depressed ulcerative gastric lesion. Histopathological examination confirmed poorly differentiated gastric adenocarcinoma. Intraoperative biopsy of retroperitoneal fibrotic tissue showed malignant cell infiltration, confirming RPF secondary to gastric cancer.

**Interventions:**

The patient underwent subtotal gastrectomy and bilateral percutaneous nephrostomy for urinary diversion. Antibiotics were administered postoperatively for urinary tract infection. She was discharged with a plan for adjuvant chemotherapy.

**Outcomes:**

Urine output and renal function normalized following percutaneous nephrostomy insertion. The patient’s infection resolved with antibiotic therapy, and she remained clinically stable at discharge.

**Lessons:**

This case illustrates a rare presentation of gastric cancer manifesting as malignancy-associated RPF. Clinicians should maintain a high index of suspicion for occult malignancy in patients with bilateral ureteral obstruction of unclear cause. Early imaging and histological evaluation are critical for diagnosis and appropriate management.

## 1. Introduction

Retroperitoneal fibrosis (RPF) is a rare fibroinflammatory disorder characterized by excessive collagen deposition and chronic inflammation in the retroperitoneal space, often leading to ureteral obstruction, vascular compression, and various systemic complications.^[1,2]^ RPF has an estimated annual incidence of 1.3 per 100,000 persons.^[2,3]^ Approximately 70% of cases are idiopathic, while 8% are attributed to malignancies. While idiopathic cases account for the majority of RPF, secondary causes – including malignancies, infections, medications, and autoimmune diseases – must be considered. Among malignancy-related RPF, lymphoma,^[4]^ sarcoma,^[5]^ and metastatic carcinomas^[5]^ have been more frequently reported, but its association with primary gastric cancer remains exceptionally rare.^[6,7]^

Gastric cancer is one of the most prevalent malignancies worldwide, with common metastatic sites including the liver, peritoneum, and lymph nodes. However, its involvement in the retroperitoneal space leading to fibrotic reactions is not well-documented in the literature. The pathophysiology underlying malignancy-associated RPF remains unclear, but proposed mechanisms include direct tumor invasion, immune-mediated responses, and tumor-induced fibroblast activation.^[1,8]^ Given the rarity of this condition, recognizing gastric cancer as a potential etiology of RPF is crucial for timely diagnosis and appropriate management.

Here, we report a rare case of RPF secondary to gastric cancer, emphasizing its clinical presentation, diagnostic challenges, and therapeutic implications. This case highlights the need for clinicians to consider RPF in the differential diagnosis of unexplained retroperitoneal masses or ureteral obstruction in patients with gastric cancer.

## 2. Case presentation

**Figure 1.**
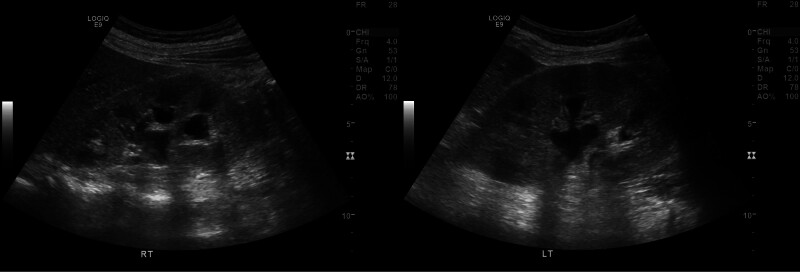
Abdominal ultrasound showing both kidneys of normal size (right: 11.26 cm, left: 11.36 cm) with slightly increased cortical echogenicity and bilateral hydronephrosis.

On Day 1, a 56-year-old woman presented to a nephrologist with complaints of generalized edema and decreased urine output that had begun 7 days prior. She also reported nausea following the reduction in urine output, along with a loss of appetite. Renal ultrasound revealed bilateral hydronephrosis and slightly increased echogenicity in both kidneys (Fig. 1). She had no significant past medical or family history.

At the time of admission, her vital signs were stable: blood pressure, 142/76 mm Hg; pulse rate, 79 beats per minute; respiratory rate, 20 breaths per minute; and body temperature, 36.6°C. Initial laboratory tests showed elevated creatinine levels (1.51 mg/dL) and blood urea nitrogen (BUN) of 23 mg/dL. She was found to be anemic, with a hemoglobin level of 9.8 g/dL. Her white blood cell count was within the normal range at 4.56 × 10⁹/L (reference range: 4.0–10.0 × 10⁹/L), and her C-reactive protein level was 0.2 mg/dL (reference range: 0–0.5 mg/dL). The erythrocyte sedimentation rate was 14 mm/h (reference range: 0–20 mm/h). Procalcitonin was slightly elevated at 0.073 ng/mL (reference range: <0.046 ng/mL). Urinalysis revealed no proteinuria, hematuria, or pyuria. The urine albumin-to-creatinine ratio was 10.7 mg/g (reference range: <30 mg/g). Tumor markers, including carcinoembryonic antigen, carbohydrate antigen 19-9, and carbohydrate antigen 125, were all within normal limits. To evaluate for immunoglobulin G subclass 4 (IgG4)-related disease as a possible cause of RPF, serum total immunoglobulin G (IgG) level was measured at 965 mg/dL, and the IgG4 level was 39.1 mg/dL – both within normal limits.

On Day 2 and 3, despite diuretic administration, urine output did not improve, and creatinine levels progressively increased to 5.27 mg/dL, with persistent bilateral hydronephrosis. While the rise in creatinine was significant, the increase in BUN was comparatively mild, reaching 25 mg/dL. On Day 4, bilateral double J stents were inserted following consultation with a urologist. However, even after stent placement, laboratory parameters did not improve, and urine output continued to decline. On Day 5, consequently, bilateral percutaneous nephrostomy (PCN) catheters were inserted. Following PCN placement, urinary drainage improved, and both creatinine and BUN levels normalized.

**Figure 2.**
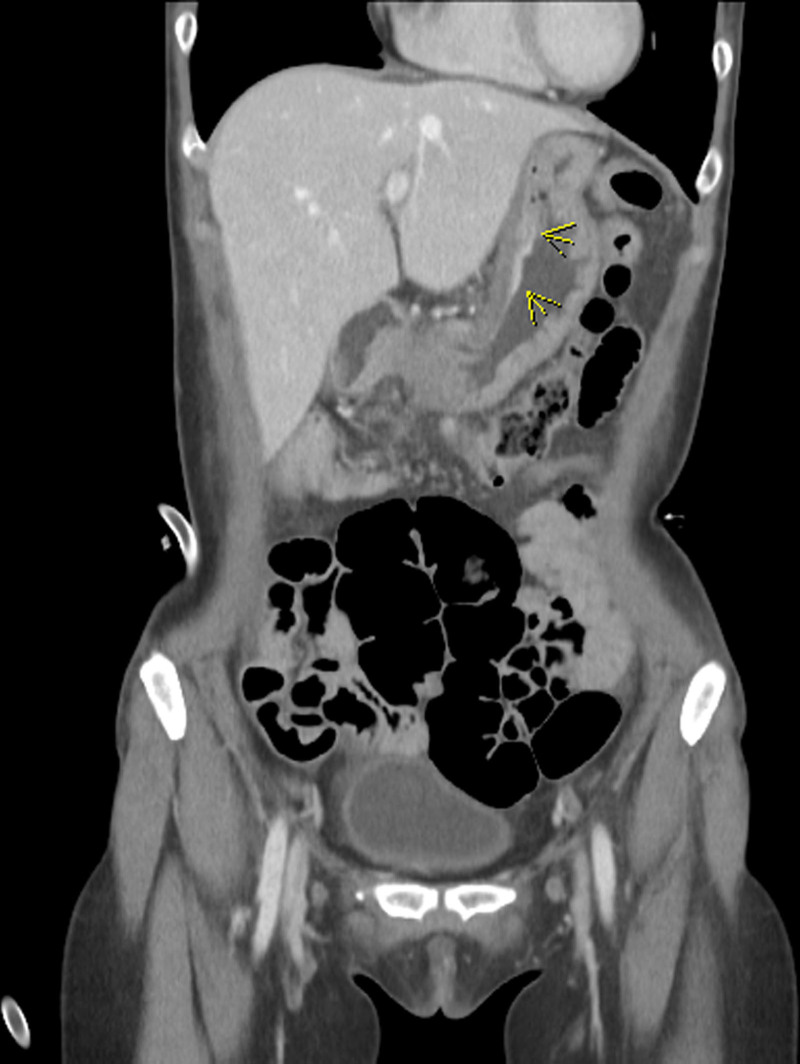
Abdominal-pelvic computed tomography (APCT) showing edematous wall thickening and suspicious mucosal hyperenhancement at the lesser curvature side of the gastric body (yellow arrow).

**Figure 3.**
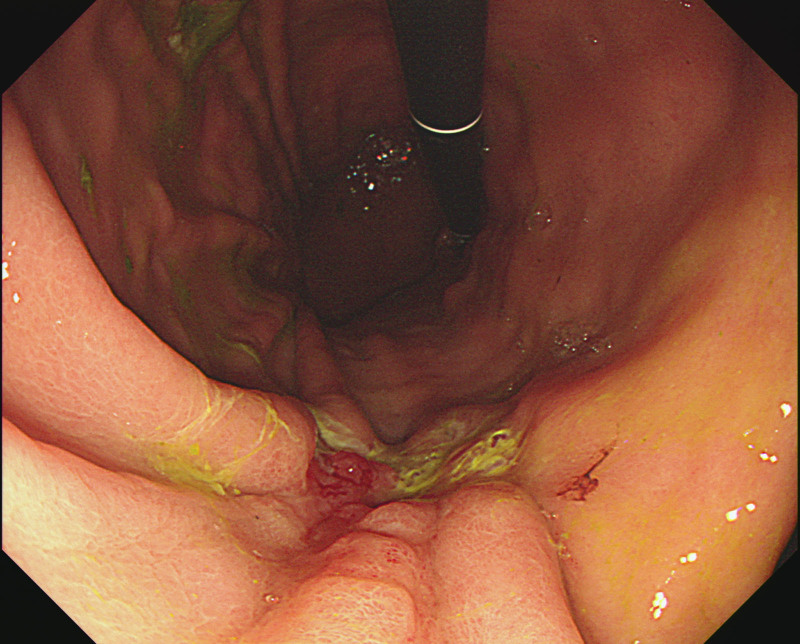
Esophagogastroduodenoscopy (EGD) revealing a 25-mm lesion with central ulceration, irregular margins, and fold convergence on the anterior wall of the lower body near the lesser curvature.

On Day 6, to investigate the underlying cause of bilateral hydronephrosis, an abdominal-pelvic computed tomography was performed. The scan revealed edematous wall thickening and mucosal hyperenhancement of the gastric body (Fig. 2), though no significant enlargement of peripheral lymph nodes was observed. On Day 8, subsequent esophagogastroduodenoscopy identified a 25-mm depressed and ulcerative lesion on the anterior wall of the lower gastric body (Fig. 3). The lesion exhibited fold fusion and features highly suggestive of muscle invasion based on gross morphology. Histopathological examination of biopsy specimens confirmed a diagnosis of poorly differentiated adenocarcinoma. On Day 11, the patient was subsequently referred to the surgical department and underwent subtotal gastrectomy.

During surgery, extensive fibrosis of the gastric wall and retroperitoneum was macroscopically evident. Intraoperative pathology examination also revealed fibrosis in a portion of the omentum. Biopsies of retroperitoneal tissue and omental tissue were performed, along with cytopathological examination of pelvic ascitic fluid. On Day 14, final pathological findings confirmed poorly differentiated tubular adenocarcinoma with serosal invasion. No tumor involvement was detected at the resection margins; however, lymphatic, neural, and vascular invasion were all present. Metastasis was observed in 37 of 46 regional lymph nodes. While no tumor cells were identified in the omentum, malignancy was confirmed in the retroperitoneal tissue.

On Day 15 and 16, following surgery, the double J stents were removed, and the patient continued to have both PCNs in place. On postoperative day 4 (Day 17), the patient developed a high fever (up to 38.9°C), and laboratory markers of infection increased. Urine culture identified *Klebsiella pneumoniae* without extended-spectrum beta-lactamase production. Given the clinical presentation and laboratory findings, a urinary tract infection was suspected as the cause of the fever. From Day 18 to 20, the patient was treated with intravenous antibiotics, leading to fever resolution and normalization of infection markers. On Day 21, after clinical improvement, she was discharged with plans for regular PCN replacement and additional chemotherapy.

## 3. Discussion

RPF is an uncommon disease process characterized by the proliferation of inflammatory and fibrous tissue around the infrarenal abdominal aorta and iliac arteries, often encasing adjacent structures such as the ureters. Clinically, this fibrotic reaction can lead to ureteral compression, resulting in bilateral hydronephrosis, obstructive uropathy, and renal dysfunction. Presenting symptoms are often nonspecific, including abdominal or flank pain, lower extremity edema, and constitutional symptoms such as anorexia or fatigue.^[9,10]^

The differential diagnosis of RPF is broad. Approximately 70% of cases are idiopathic, while 8% are attributed to malignancies.^[1,3,11,12]^ Secondary causes include prior abdominal or pelvic surgery, radiation therapy, trauma, infections, and autoimmune or inflammatory diseases. Malignancy-associated RPF is most commonly seen with carcinoid tumors (via serotonin-mediated pathways), Hodgkin and non-Hodgkin lymphomas, sarcomas, and carcinomas of the colon, bladder, prostate, and breast.^[7,13,14]^ These tumors can induce RPF through desmoplastic reactions or tumor-derived fibrogenic cytokines.^[1,15]^

Given the patient’s presentation with bilateral ureteral obstruction and retroperitoneal soft tissue thickening, several differential diagnoses were considered, including idiopathic RPF, IgG4-related disease, malignancy-associated RPF, and other inflammatory or infectious conditions.

Laboratory findings revealed normal levels of total IgG (965 mg/dL) and IgG subclass 4 (39.1 mg/dL), making IgG4-related disease unlikely. There was no history of prior abdominal or pelvic surgery, radiation therapy, trauma, or systemic autoimmune disease to support other secondary causes.

Imaging revealed gastric wall thickening, and subsequent endoscopy with biopsy confirmed poorly differentiated gastric adenocarcinoma. Intraoperative biopsies of retroperitoneal tissue demonstrated malignant cell infiltration, which provided definitive evidence of RPF secondary to gastric cancer.

These findings support a direct neoplastic etiology for the RPF rather than idiopathic or immune-mediated causes.

The pathogenesis of malignancy-related RPF may involve several mechanisms, including direct tumor invasion – as demonstrated in our case – desmoplastic responses to cytokines such as transforming growth factor-β, and paraneoplastic autoimmune processes.^[1]^

Management of malignancy-associated RPF differs significantly from that of idiopathic disease. Immunosuppressive therapies, often effective in idiopathic cases, are insufficient for tumor-related fibrosis and may delay cancer treatment. Therefore, identifying the underlying etiology is essential to guide management. In this case, the patient underwent subtotal gastrectomy, PCN for urinary diversion, and was planned for systemic chemotherapy.

To our knowledge, this is one of few reported cases of pathologically confirmed RPF secondary to gastric adenocarcinoma. This case highlights the diagnostic challenges of RPF and emphasizes the need to consider underlying malignancy when evaluating bilateral ureteral obstruction of unclear etiology.

In conclusion, although rare, gastric cancer should be recognized as a potential cause of RPF. In cases of bilateral ureteral obstruction with no identifiable secondary cause, clinicians should maintain a high index of suspicion for occult malignancy. Evaluation for IgG4-related disease should also be considered as part of the initial diagnostic workup for RPF. Timely imaging, histological confirmation, and interdisciplinary management are essential for optimizing outcomes in malignancy-associated RPF.

## Author contributions

**Conceptualization:** Su Bee Park.

**Resources:** Su Bee Park.

**Supervision:** Su Bee Park.

**Validation:** Moon Hyung Lee.

**Visualization:** Jiwon Kim.

**Writing – original draft:** Jiwon Kim, Moon Hyung Lee, Su Bee Park.

**Writing – review & editing:** Jiwon Kim, Moon Hyung Lee, Su Bee Park.
